# MFP-FePt-GO Nanocomposites Promote Radiosensitivity of Non-Small Cell Lung Cancer Via Activating Mitochondrial-Mediated Apoptosis and Impairing DNA Damage Repair

**DOI:** 10.7150/ijbs.46194

**Published:** 2020-05-18

**Authors:** Shan Peng, Yingming Sun, Yuan Luo, Shijing Ma, Wenjie Sun, Guiliang Tang, Shuying Li, Nannan Zhang, Jiangbo Ren, Yu Xiao, Xuefeng Liu, Junhong Zhang, Yan Gong, Conghua Xie

**Affiliations:** 1 Department of Radiation and Medical Oncology, Zhongnan Hospital of Wuhan University, Wuhan, China; 2 Department of Biological Repositories, Zhongnan Hospital of Wuhan University, Wuhan, China; 3 Department of Pathology, Lombardi Comprehensive Cancer Center Georgetown University Medical School, Washington DC, USA; 4 Hubei Key Laboratory of Tumor Biological Behaviors, Zhongnan Hospital of Wuhan University, Wuhan, China; 5 Hubei Cancer Clinical Study Center, Zhongnan Hospital of Wuhan University, Wuhan, China

**Keywords:** MFP-FePt-GO nanocomposites, radiosensitivity, non-small cell lung cancer, apoptosis, DNA damage repair

## Abstract

**Background**: Recent advances in nanomedicine provided promising alternatives for tumor treatment to improve the survival and life quality of cancer patients. This study was designed to explore the insight mechanisms of the anti-tumor effects of the novel nanocomposites (NCs) MFP-FePt-GO with non-small cell lung cancer (NSCLC).

**Methods**: A chemical co-reduction method was applied to the synthesis process of MFP-FePt-GO NCs. The chemical synthesis efficiency and morphology of the NCs were measured with spectroscope and transmission electron microscope. Colony formation assay and cell apoptosis were conducted to assess the radiosensitivity effect of NCs with radiation. Then, we detected cell mitochondrial membrane potential and reactive oxygen species (ROS) level by flow cytometry to further explore the cause of cell death. Immunofluorescence staining and Confocal were carried out to determine the DNA damage repair. A Lewis lung carcinoma animal model was used to measure safety and anti-tumor efficiency *in vivo*.

**Results**: The novel NCs MFP-FePt-GO designed on a lamellar-structure magnetic graphene oxide and polyethylene glycol drug delivery system was synthesized and functionalized for co-delivery of metronidazole and 5-fluorouracil. While no severe allergies, liver and kidney damage, or drug-related deaths were observed, MFP-FePt-GO NCs promoted radiosensitivity of NSCLC cells both *in vivo* and i*n vitro*. It improved the effects of radiation via activating intrinsic mitochondrial-mediated apoptosis and impairing DNA damage repair. This NCs also induced a ROS burst, which suppressed the antioxidant protein expression and induced cell apoptosis. Furthermore, MFP-FePt-GO NCs prevented NSCLC cell migration and invasion.

**Conclusion**: MFP-FePt-GO NCs showed a synergistic anti-tumor effect with radiation to eliminate tumors. With good safety and efficacy, this novel NCs could be a potential radiosensitive agent for NSCLC patients.

## Introduction

Non-small cell Lung cancer (NSCLC) is the leading cause of cancer-related deaths among men and women worldwide, accounting for 1.8 million new cases and 1.6 million deaths in 2012 1. The 5-year survival rates of NSCLC patients varied (4-17%), relying on the stages and regional differences, strikingly lower than any other common cancer 2. Although significant advances have been made in high-risk smoker screening, diagnostic modalities, and chemo-preventive approaches, the majority of lung cancer patients are still diagnosed at a late stage. Improvements in surgery and radiotherapy advanced our ability to manage patients at the early stages, particularly the ones unlikely to benefit from traditional open resection 3. The use of small molecule tyrosine kinase inhibitors and immunotherapy led to unprecedented survival benefits in selected patients during the last decades. However, the overall cure and survival rates of NSCLC remain low. Therefore, continuous studies on novel drugs and combined therapies are required to expand the clinical benefit to a more extensive population and to improve NSCLC prognosis 4.

The development of nanomaterials substantially improved tumor diagnosis and treatment 5. As an inorganic nanomaterial, graphene nanocomposites (NCs) gained more attention in cancer nanotechnology compared with other inorganic nanomaterials recently 6. Graphene NCs were extensively explored as potential chemotherapeutic carriers and theranostic tools due to its numerous physicochemical properties, including multiple pay load capability, drug targeting functions and photothermal effects 6. Furthermore, introduction of various reactive functional groups on the surface of nanomaterials allows us to conjugate a spectrum of contrast agents, antibodies, peptides, ligands and drugs and, and to construct multifunctional and hybrid nanomaterials for the visualization and treatment of cancers 7. With the development of nano-formulations and nanoparticle-based drug delivery, researchers explored dual delivery of biological therapeutics to overcome the current drawbacks of cancer therapy. Compared with the conventional single drug therapy, dual delivery of therapeutics provides synergistic effects in addition to treatment multimodality 8.

Graphene oxide (GO) is the oxidation derivative of graphene. This 2-dimensional lamellar structure nanosystem is only one-atom thick 9, 10, characterized by expanded surface area and excellent biocompatibility 11. Moreover, PEGylated GO was reported to efficiently avoid the aggregation risk in solutions with high salts and proteins 12. FePt nanoparticles (NPs) present substantial advantages in biomedical applications due to its optimal magnetic, optical properties and controllable morphology 13, 14. Magnetic NPs can be collected and manipulated by an external magnetic field, promoting the accumulation of NPs to the targeted lesions 15. Moreover, nitroimidazole, including metronidazole, was reported to facilitate radical anions production, as the key element to cause DNA damage during radiation 16. However, the application of nitroimidazole in clinic is limited by its high toxicity 17. Therefore, the predicable solution of its employment as a radiosensitizer involves transporting nitroimidazole into tumor area by a nano drug delivery system and minimizing the normal tissue cytotoxicity 18. In addition, 5-fluorouracil (5-FU) was applied as deoxy thymidylate synthetase inhibitor in concurrent chemoradiotherapy to impair DNA synthesis in lung cancer treatment.

In our study, the novel MFP-FePt-GO NCs were designed based on a lamellar-structure magnetic GO and polyethylene glycol drug delivery system. MFP-FePt-GO NCs were synthesized and functionalized for co-delivery of metronidazole and 5-FU. The anti-tumor effects of MFP-FePt-GO NCs were explored in NSCLC cells *in vitro* and *in vivo*.

## Materials and Methods

### The synthesis of MFP-FePt-GO NCs

**Figure 1:**
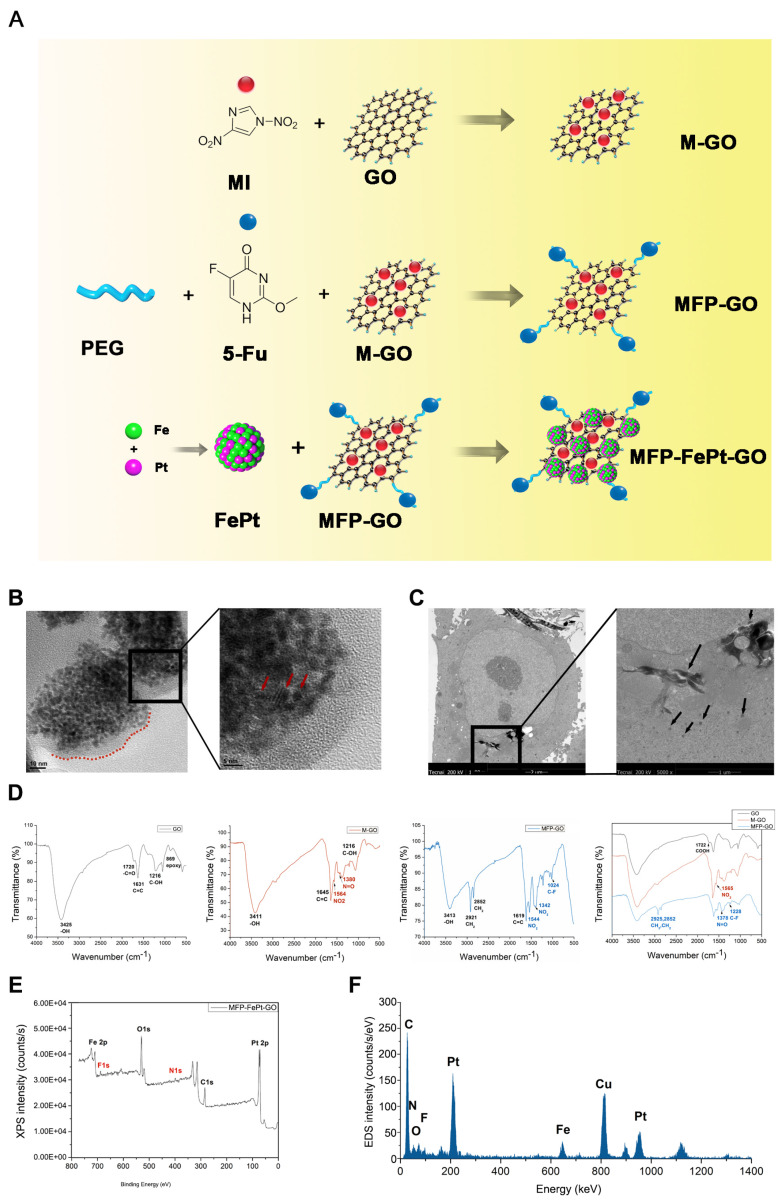
** The synthesis process and physical characterization of MFP-FePt-GO NCs. (A)** The synthesis process of MFP-FePt-GO NCs. MI, metronidazole. GO, graphene oxide. PEG, polyethylene glycol. 5-Fu, 5-fluorouracil. **(B)** Scanning electron microscope graphs of MFP-FePt-GO NCs in scale of 10 nm (left panel) and 5 nm (right panel). The lamellar structure of GO is shown in the left panel labelled with a red dotted line. In the right panel, the spherical FePt particles indicated by the red arrows were deposited uniformly on the GO surface at a high loading rate. **(C)** TEM images of cells that phagocytose MFP-FePt-GO NCs suggest the uptaken of lamellar-structure NCs. The arrows indicate the released FePt NPs in the cytoplasm. GO and mental particles, rather than other compounds, are visualized in the TEM images. The black flakes are GO nanocomposites, and the individual dark dots are FePt NPs. **(D)** The Fourier-transform infrared spectroscopy spectra of GO, M-GO and MFP-GO. **(E)** The physical characteristics of MFP-FePt-GO detected by XPS. **(F)** EDS analysis of MFP-FePt-GO. Representative images are shown.

A chemical co-reduction method was applied to the synthesis process of MFP-FePt-GO NCs (Fig. 1A) 14, 19. Polyethylene glycol 2000 (PEG, 500 mg, Sinopharm, China) was dissolved in 100 mL deionized water. GO (25 mg, Suzhou Tanfen, China), 1-Ethyl-3-(3-dimethylaminopropyl) carbodiimide (668 mg, Shanghai Medpep, China), and 2-(2-methyl-5-nitro-1H-imidazole-1-yl) (MI, 560 mg, Shanghai Bioengineering, China) were added and mixed thoroughly. Dimethylaminopyridin (854 mg, Shanghai Titan, China) was then added slowly at room temperature (RT). The mixtures were stirred vigorously for 72 h. The brownish products obtained by centrifugation were MP-GO NCs.

Iron acetylacetonate (98%, 0.386 mmol, Aladdin, USA), oleic acid (1.5 mL, Aladdin) and oleylamine (95%, 1.5 mL, Aladdin) were sequentially added into a flask with 100 mL anhydrous ethanol. After stirring for 30 min, chloroplatinic acid (19.3 mM, 20 mL, Shanghai Chemical Reagent, China) was added dropwise, and stirred for another 30 min. Sodium borohydride (65.7 mM, 200 mL, Sinopharm) was then added dropwise at 40°C and stirred for 1 h. The black sediments (FePt NPs) were separated by centrifugation and dried in a vacuum oven at 40°C overnight.

FePt NPs (50 mg) and MP-GO NCs (80 mg) were dissolved in 150 mL deionized water. 5-FU (20 mL, 20 mg/mL, Tianjin Xinyao, China) was added to the mixture. The mixture was sonicated at RT for 6 h, and the black product (MFP-FePt-GO NCs) was obtained by centrifugation and dried in vacuum at 40°C overnight.

### Physical characterization of MFP-FePt-GO NCs

The size, morphology, and dispersibility of MFP-FePt-GO NCs were measured on a scanning electron microscope (JEM2010FEF-Ω, JEOL, Japan). The uptaken of lamellar-structure NCs by cells was examined by a 200 KV transmission electron microscope (TEM, Tecnai G20 TWIN, FEI, USA). The chemical structure was verified by Fourier transform infrared spectroscope (Nicolet 6700, Thermo Fisher, USA). The surface chemical elements were analyzed by X-ray photoelectron spectroscopy (XPS) and energy dispersive X-ray spectroscopy (EDS, ESCALAB250Xi, Thermo Fisher, USA).

### Cells

The BEAS-2B (human lung epithelial cell), A549 (human lung adenocarcinoma cell), H1975 (human lung adenocarcinoma cell) and Lewis lung carcinoma (LLC, mouse lung cancer cell) cell lines were purchased from the Type Culture Collection of the Chinese Academy of Sciences (Shanghai, China). A549 and H1975 were cultured in RPMI-1640 medium (HyClone, USA), and BEAS and LLC were cultured in DMEM (HyClone), both with 10% fetal bovine serum (FBS, HyClone), penicillin and streptomycin (HyClone) at 37 ºC with 5% CO_2_.

### Cell viability assay

Cells were seeded in 96-well plates, cultured for 24 h, and treated with MFP-FePt-GO NCs at different concentrations (1, 10, 20, 30, 40, 50, 60, 70, 80, 90, 100 μg/mL) for 24 h. Two hours after addition of CCK8 (10 μL, Dojindo, Japan) into each well at 37 ºC, the absorbance at 450 nm was measured by the Rayto-6000 system (Rayto, China). Cell viability = (OD value of treated cells - OD value of medium blank control) / (OD value of untreated cells - OD value of medium blank control) × 100%

### Colony formation assay for radiosensitivity

The NSCLC cells were seeded in 6‐well plates and cultured overnight. Six hours after addition of MFP-FePt-GO NCs (10 μg/mL), the cells were irradiated at doses of 0, 1, 2, 4, 6, 8 and 10 Gy. The colonies were fixed and stained 14 days later. The numbers of colonies were counted by 3 different investigators. A “multi-target single-hit model" was applied to fit the survival curve 20.

### Flow cytometry

The cells were harvested and stained with FITC-conjugated annexin V (BD, USA) and propidium iodide (BD, USA). The intracellular relative oxygen species (ROS) accumulation was measured as previously described 21. The cells were stained with DCFH-DA (10 μM, Beyotime, China). The Mitochondrial membrane potential detection kit (Beyotime, China) was used for JC-1 staining. The stained cells were assessed by flow cytometry (FACS Aria III, BD, USA).

### Cell apoptosis assay

The cells cultured on 24 mm coverslips. TUNEL assay was performed according to the manufacturer's instruction (Roche, Germany). The nuclei were stained with DAPI, and the staining was analyzed by the fluorescent microscope (IX 73 DP80, Olympus, Japan). The positive cells were count as previous described 22.

### Wound healing assay

The cells were seeded in 12-well plates and grown to confluent monolayer. The monolayer was scratched straight with a 10 μL pipette tip. Migration was visualized at the indicated times (0, 24 and 48 h). The migration distances were measured by ImageJ analysis software (NIH, Bethesda, MD).

### Modified Boyden chamber invasion assay

The 8‐μm diameter filters (Corning, USA) for 24‐well plates were precoated with Matrigel (40 μL, Corning). Approximately 1 × 10^5^ cells in 200 μL of serum‐free RPMI‐1640 medium were placed in the upper chamber, and 500 μL RPMI‐1640 medium with 10% FBS was placed in the lower chamber. The cells on the underside of the filters were examined and counted under a microscope (DC 300F, Leica, Germany) 24 h later.

### Immunoblotting analysis

The cells were lysed in RIPA buffer containing protease inhibitor and phosphatase inhibitor (Sigma, USA). The total protein was separated using 7.5-12.5% SDS-PAGE (Bio-Rad, USA) and transferred to a PVDF membrane (Millipore, USA). The primary antibodies Caspase 8, PARP, BCL2, γ-H2AX, p62, pP53, E-cadherin and Vimentin were purchased from Cell Signaling Technology, USA. Other primary antibodies GAPDH, BAX, p53, RAD51, GADD45A, Histone-H3, N-cadherin, SOD2 and Catalase were purchased from Proteintech Group, USA. The immunoreactive proteins were detected by chemiluminescence (Bio-Rad).

### Immunofluorescent assay

The cells cultured on cover slides fixed with PFA, penetrated with Triton X-100 and blocked in 5% bovine serum albumin at RT for 1 h. After incubated with primary antibody at 4 ºC overnight, the slides incubated with Cy3-labelled or FITC-labelled secondary antibody at RT for 1 h. The nuclei were labeled with DAPI, and the staining was analyzed by IX 73 DP80 fluorescent microscope (Olympus) and laser confocal microscope (C2, Nikon, Japan). The mean density was applied to semi-qualified by Image-Pro Plus 6.0.

### Animals

Six-week-old female *C57BL/6* mice were purchased from the Center for Disease Control and Prevention of Hubei Province (Wuhan, China). All animal experiments were performed according to the National Institutes of Health guidelines and Wuhan University Animal Using Protocol, and were approved by the Institutional Animal Care and Use Committee (IACUC) at Wuhan University.

### Xenograft tumor growth in vivo assay

LLC cells were transfected with luciferase labeled-lentivirus according to previous publication for the visualization of tumor volumes. Approximately 5 × 10^6^ Luciferase-LLC cells were injected subcutaneously into the left groin of each mouse. Treatment was commenced when the tumor size reached ~100 mm^3^. The mice were randomized into 4 groups (6 mice per group) as follows: Saline as an untreated control, MFP-FePt-GO NCs (20 μg intratumor injection every other day for 1 week), radiation (24 Gy / 3 F, small animal irradiation research platform, X-RAD 225Cx, PXI, USA), MFP-FePt-GO NCs combined with radiation. The size of the tumors and the weight of the mice were recorded every other day. Tumor volume (V) was calculated as (length × width^2^)/2. The mice were sacrificed when tumor size reached 1800 mm^3^, tumor influenced breathing, eating, walking and any other physiologic functions, or tumor surface appeared anabrosis. The size of tumors *in vivo* was measured by the IVIS Lumina XRMS Series III system with luciferase-mediated bioluminescence. The blood samples were collected as previous protocols 23. The levels of alanine transaminase (ALT), aspartate aminotransferase (AST) and blood urea nitrogen (BUN) were detected with ELISA kits (MyBioSource, USA).

### Statistical analyses

Each experiment was performed in triplicates and data presented in representation of 3 individual experiments. A two-tailed Student's t-test and one-way analysis of variance (ANOVA) were used to evaluate the statistical significance of different groups. Statistical analyses were performed with SPSS 16.0. P values < 0.05 were considered as statistical significance.

## Results

### Characterization of MFP-FePt-GO NCs

The chemical synthesis process of MFP-FePt-GO NCs is shown in Fig. 1A. The morphology, dispersion, and size of MFP-FePt-GO NCs were characterized by scanning electron microscopy (Fig. 1B). The TEM images indicated that cells phagocytosed MFP-FePt-GO NCs (Fig. 1C), suggesting the uptake of lamellar-structure NCs by H1975. The arrows in Fig. 1C indicated the released FePt nanocomposites in the cytoplasm. In the spectrum of GO, the strong and broad peak at 3425 cm^-1^ (-OH) increased from the stretching vibration of the N-H bond due to hydroxyls and undried water molecules (Fig. 1D). The dispersibility of NCs was markedly improved in aqueous solutions, especially in cell culture medium. The signal peak at 1720 cm^-1^ (-C=O) indicated the presence of the carbonyls in the carboxyl groups (Fig. 1D) 24. In the spectra of GO-M, the characteristic peaks at 1564 cm^-1^ (NO_2_) and 1380 cm^-1^ (N=O) indicated the binding of nitro groups of MI (Fig. 1D). In the spectra of GO-MFP-FePt NCs, the successful load of 5-FU on GO-MI NPs was verified by the enhanced characteristic peaks of methylene, which located at 2921 cm^-1^ (CH_2_) and 2852 cm^-1^ (CH_2_), and the appeared peak of Carbon-Fluorine bond (C-F) at 1024 cm^-1^ (Fig. 1D). No significant peaks disappeared, suggesting that 5-FU was loaded on GO via physisorption with the support of PEG, instead of a chemical bond connection 24, 25.

The XPS and EDS were carried out to define the chemical composition of the NCs 26, 27. The XPS spectra of MFP-FePt-GO showed the peak of binding energy at 399.46 eV belongs to N1s (Fig. 1E). Moreover, the F1s peaks at the binding energy of 678.96 eV demonstrated that 5-FU was loaded, the same as Fe and Pt peaks for FePt NPs. The EDS analysis demonstrated the same conclusion, except for the peak of Cu comes from the copper mesh that load samples (Fig. 1F). These results indicated the successful synthesis of MFP-FePt-GO NCs.

### MFP-FePt-GO NCs enhanced radiosensitivity of NSCLC cells in vitro

**Figure 2:**
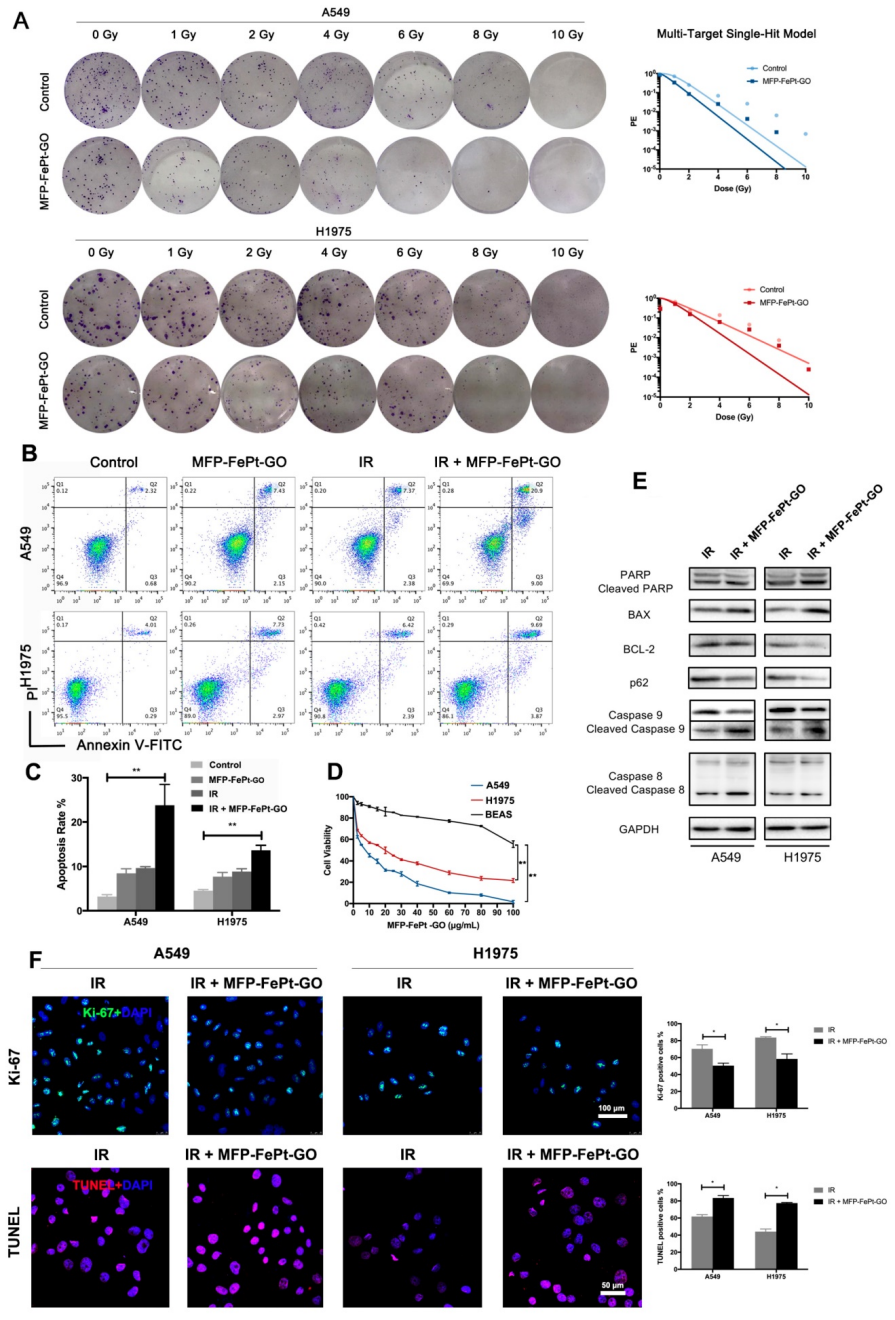
** MFP-FePt-GO enhances NSCLC cell radiosensitivity. (A)** Colony formation assay shows that MFP-FePt-GO (20 μg/mL) enhances radiosensitivity of NSCLC cells *in vitro*, and cell survival fraction is evaluated by radiation multi-target single-hit model. **(B)** and **(C)** MFP-FePt-GO increases NSCLC cell apoptosis induced by IR (4 Gy). **(D)** The cell viability of A549, H1975 and BEAS treated with MFP-FePt-GO. **(E)** Representative immunoblotting of PARP, BAX and BCL-2, p62, Caspase 9 and Caspase 8 in lung cancer cells. **(F)** Representative Ki-67 and TUNEL staining images in NSCLC cells suggest that MFP-FePt-GO inhibits cell proliferation and induces cell apoptosis. *, p < 0.05; **, p < 0.01.

To investigate the synergistic effects of MFP-FePt-GO NCs on radiosensitivity of NSCLC cells *in vitro*, the long-term cell killing effects of radiation were examined by the colony formation assay. With a gradient dose of irradiation, MFP-FePt-GO NCs reduced colony numbers in each sub-group (Fig. 2A). Moreover, MFP-FePt-GO NCs increased NSCLC cell apoptosis induced by radiation (Fig. 2B and 2C). The cell viability indicated that MFP-FePt-GO NCs inhibited NSCLC cell growth (Fig. 2D).

Interestingly, our results suggested that MFP-FePt-GO NCs had a selective effect on NSCLC cells rather than normal bronchial epithelial cells. Oxidative stress was higher in tumor cells, and the Fe2+ further stressed the cells and increased mitochondrial ROS, resulting in increased apoptosis in tumor cells. Immunoblotting results showed that irradiation combined with MFP-FePt-GO NCs increased the levels of cleaved PARP and the ratios of BAX/BCL-2, as well as cleaved caspase-9 and cleaved caspase-8 (Fig. 2E). In addition, Ki-67 staining indicated that the combination of MFP-FePt-GO NCs and radiation further attenuated NSCLC cell proliferation than the individual treatment (Fig. 2F). The TUNEL assay suggested that MFP-FePt-GO NCs treatment and irradiation had synergistic effects on NSCLC cell apoptosis. Taken together, MFP-FePt-GO NCs enhanced radiosensitivity of NSCLC cells* in vitro*.

### MFP-FePt-GO NCs induced mitochondrial dysfunction in NSCLC cells

**Figure 3:**
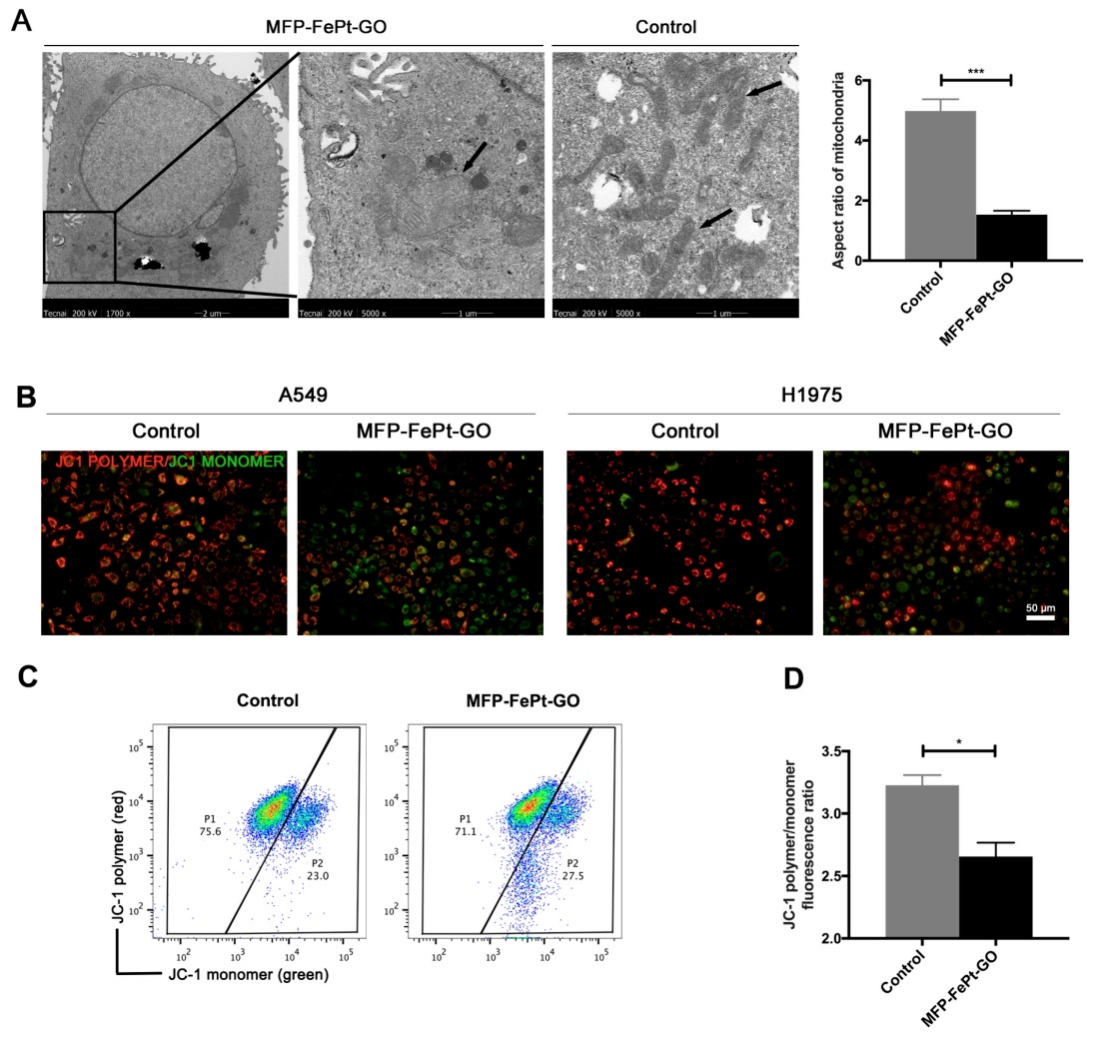
** MFP-FePt-GO induces mitochondrial dysfunction in NSCLC cells. (A)** Representative TEM images of H1975 cells treated with or without MFP-FePt-GO NCs. The morphology of mitochondrial changes from slender to swelling, and mitochondria cristae become disordered when cells phagocytose MFP-FePt-GO. The aspect ratios of mitochondria are quantified. **(B)** Confocal microscopy images of the JC-1 probe in A549 and H1975 cells shows that MFP-FePt-GO (20 μg/mL) induces the reduction of mitochondrial membrane potential (from JC-1 polymer to JC-1 monomer, from red to green). **(C)** and **(D)** Flow cytometry assay shows the quantification of ratio of JC-1 polymer/monomer of cells treated with or without MFP-FePt-GO. The results indicate the decrease of mitochondrial membrane potential with the treatment of MFP-FePt-GO. *, p < 0.05; **, p < 0.01.

To further investigate MFP-FePt-GO NCs-induced cell apoptosis, we examined NCs-induced mitochondrial changes by TEM. MFP-FePt-GO NCs phagocytosis resulted in morphology changes of mitochondria from slender to swelling and disordered mitochondria cristae (Fig. 3A). The mitochondria membrane potential was then detected using JC-1 probes. Red fluorescence indicated high mitochondrial polarization due to J-aggregate formation by the concentrated dye, while green fluorescence indicated depolarized regions. MFP-FePt-GO NCs induced the transformation of JC-1 polymer to JC-1 monomer in the mitochondria, suggesting that the cells underwent an early stage of cell apoptosis (Fig. 3B). The ratio of JC-1 polymer/monomer in cells treated with or without MFP-FePt-GO NCs demonstrated decreased mitochondrial membrane potential by MFP-FePt-GO NCs (Fig. 3C and 3D). Therefore, MFP-FePt-GO NCs increased cell apoptosis by inducing mitochondrial dysfunction in NSCLC cells.

### MFP-FePt-GO NCs increased NSCLC cell apoptosis via upregulating ROS levels and attenuating antioxidant protein expression

**Figure 4:**
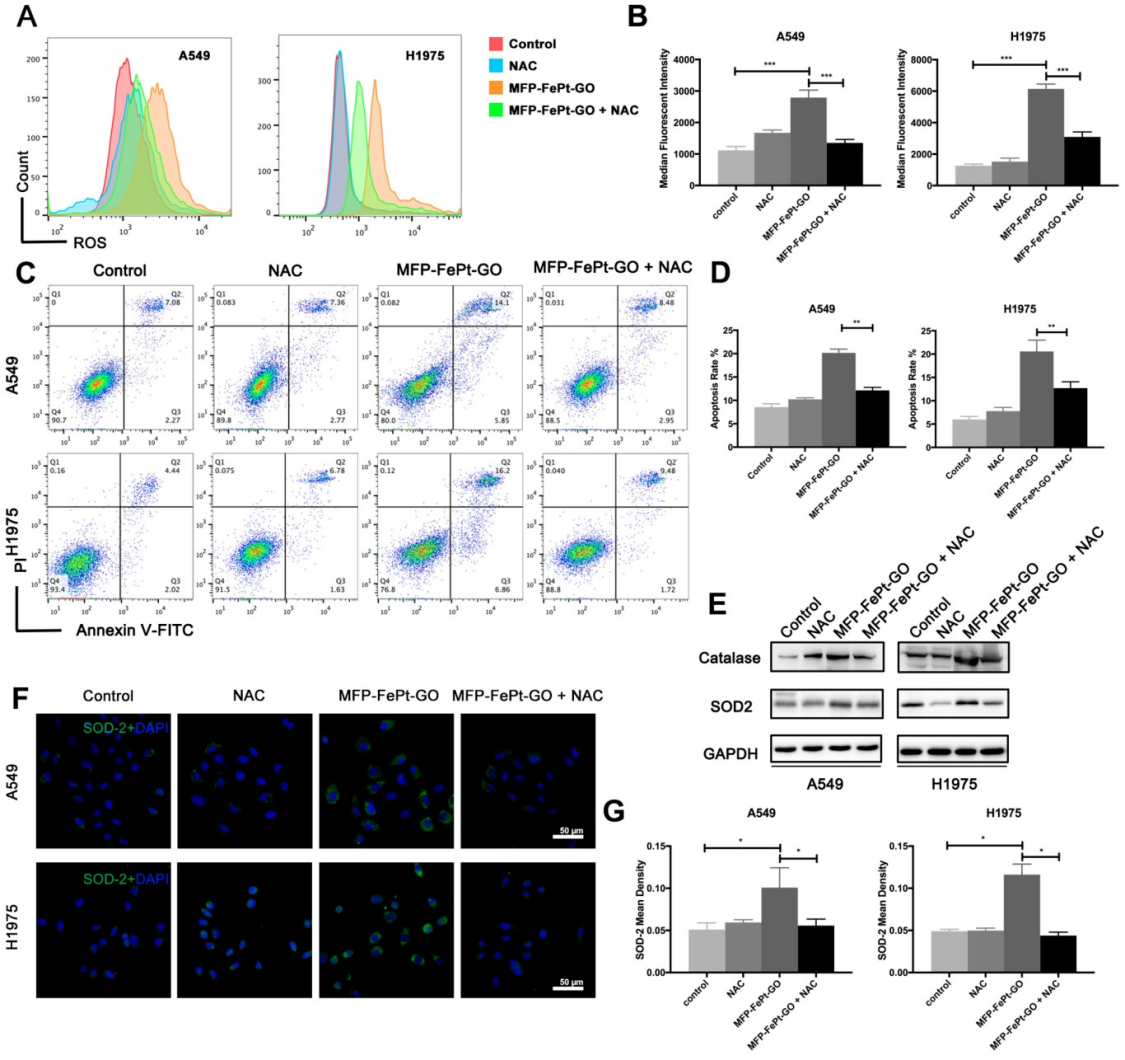
** MFP-FePt-GO increases radiosensitivity by upregulating ROS levels and attenuating antioxidant proteins expression. (A)** and **(B)** Cell cytometry analysis of ROS probe in NSCLC cells shows that NAC significantly decreases ROS levels elevated by MFP-FePt-GO (20 μg/mL). **(C)** and **(D)** Cell cytometry analysis of Annexin V and PI for apoptosis in NSCLC cells. The double treatment group has significantly more cell apoptosis than individual ones. **(E)** Representative immunoblotting of Catalase and SOD2. **(F)** and **(G)** Representative ROS probe staining shows that the combined group has significantly less ROS levels than single MFP-FePt-GO group, suggesting that MFP-FePt-GO-induced ROS can be partly blocked by NAC. *, p < 0.05; **, p < 0.01.

Apoptosis via mitochondria activates a series of downstream responses, including the ROS generation in cells 28. DCF-activated ROS and cellular apoptosis are significantly increased at 24h after MFP-FePt-GO NCs treatment (Fig. 4A and 4B). N-acetylcysteine (NAC) is an aminothiol and synthetic precursor of intracellular cysteine and glutathione, and is thus considered as an important antioxidant. The application NAC efficiently rescues the cell apoptosis induced by MFP-FePt-GO NCs (Fig. 4C and 4D). Moreover, the protein levels of catalase and SOD2 are increased in the NCs group and rescued by NAC (Fig. 4E, 4F, and 4G). These results indicate that MFP-FePt-GO NCs increased radiosensitivity by upregulating ROS levels and antioxidant proteins expression.

### MFP-FePt-GO NCs inhibited DNA damage repair in NSCLC cells

Ionizing radiation directly affects DNA structure by inducing DNA breaks, particularly, double strand breaks (DSBs). Secondary effects are the generation of ROS that oxidize proteins and lipids, and also induce single strand break (SSB). Collectively, all the changes induce cell death and mitotic failure. The radiation-sensitizing mechanism of MFP-FePt-GO NCs cannot be separated from DNA damage, so we examined its effects on DNA damage and repair.

**Figure 5:**
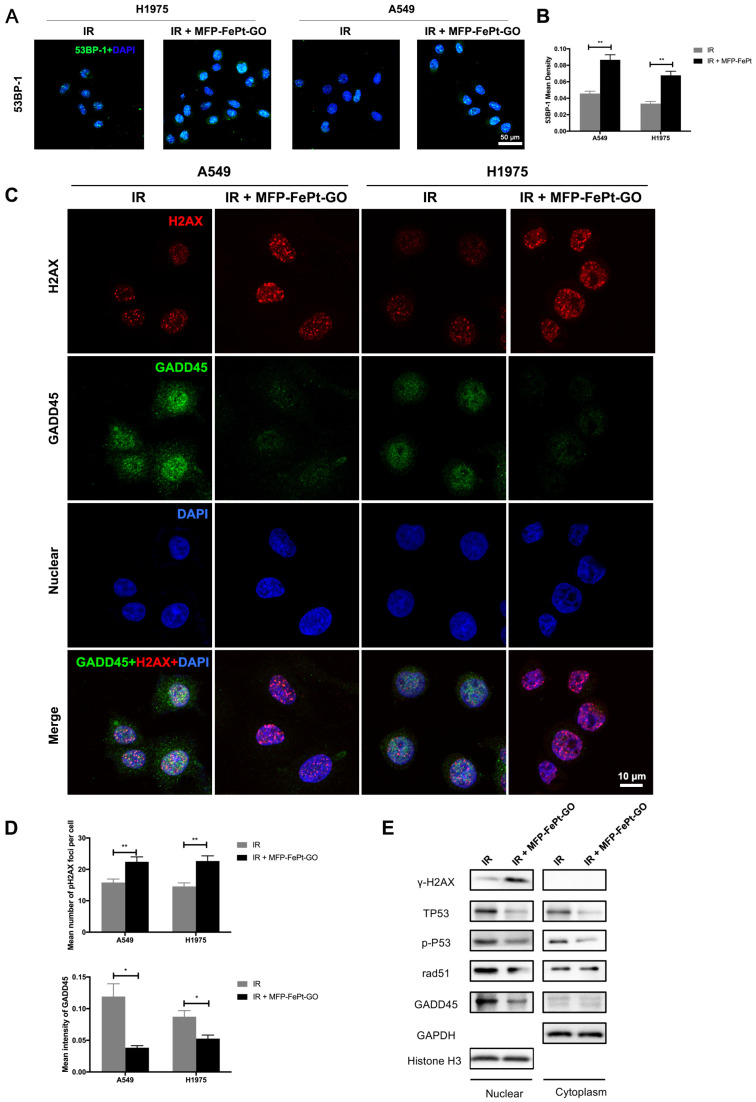
** MFP-FePt-GO increases DNA fracture by inhibiting DNA damage repair pathway. (A)** and **(B)** Representative images of 53BP-1 staining. The combined group has more 53BP-1 positive foci (green) than single radiation groups.** (C)** and **(D)** Confocal laser scanning microscope images suggest that the combination treatment of radiation (IR, 2 Gy) and MFP-FePt-GO (20 μg/mL) increases DNA fracture (γ-H2AX, red) and decreases DNA damage repair (GADD45, green).** (E)** Representative immunoblotting of DNA damage and repair-related proteins in cytoplasmic and nuclear sections. *, p < 0.05; **, p < 0.01.

The radiation-induced 53BP-1 expression was upregulated by MFP-FePt-GO NCs, suggesting increased DNA structural damages (Fig. 5A and 5B). The combination of irradiation and MFP-FePt-GO NCs increased DNA fractures (γ-H2AX, red) and decreased DNA damage repair (GADD45, green) in NSCLC cells (Fig. 5C and 5D). Immunoblotting of DNA damage and repair-related proteins in cytoplasm and nucleus suggested that irradiation combined with MFP-FePt-GO NCs upregulated γ-H2AX and downregulated p-P53, RAD-51, and GADD45, indicating increased DNA damage (Fig. 5E). These results demonstrated that MFP-FePt-GO NCs increased DNA fracture by inhibiting DNA damage repair in NSCLC cells.

### MFP-FePt-GO NCs suppressed NSCLC cell migration and invasion

The results of wound healing and transwell invasion assays indicated that MFP-FePt-GO NCs significantly impaired cell migration (Fig. S1A and S1B) and cell invasion (Fig. S1C and S1D) of both H1975 and A549 cells. Immunoblotting results showed that MFP-FePt-GO NCs upregulated E-cadherin and downregulated Vimentin and N-cadherin in both cell lines (Fig. S1E). In conclusion, MFP-FePt-GO NCs suppressed NSCLS cell migration and invasion.

### MFP-FePt-GO NCs increased radiosensitivity of lung cancer in vivo

**Figure 6:**
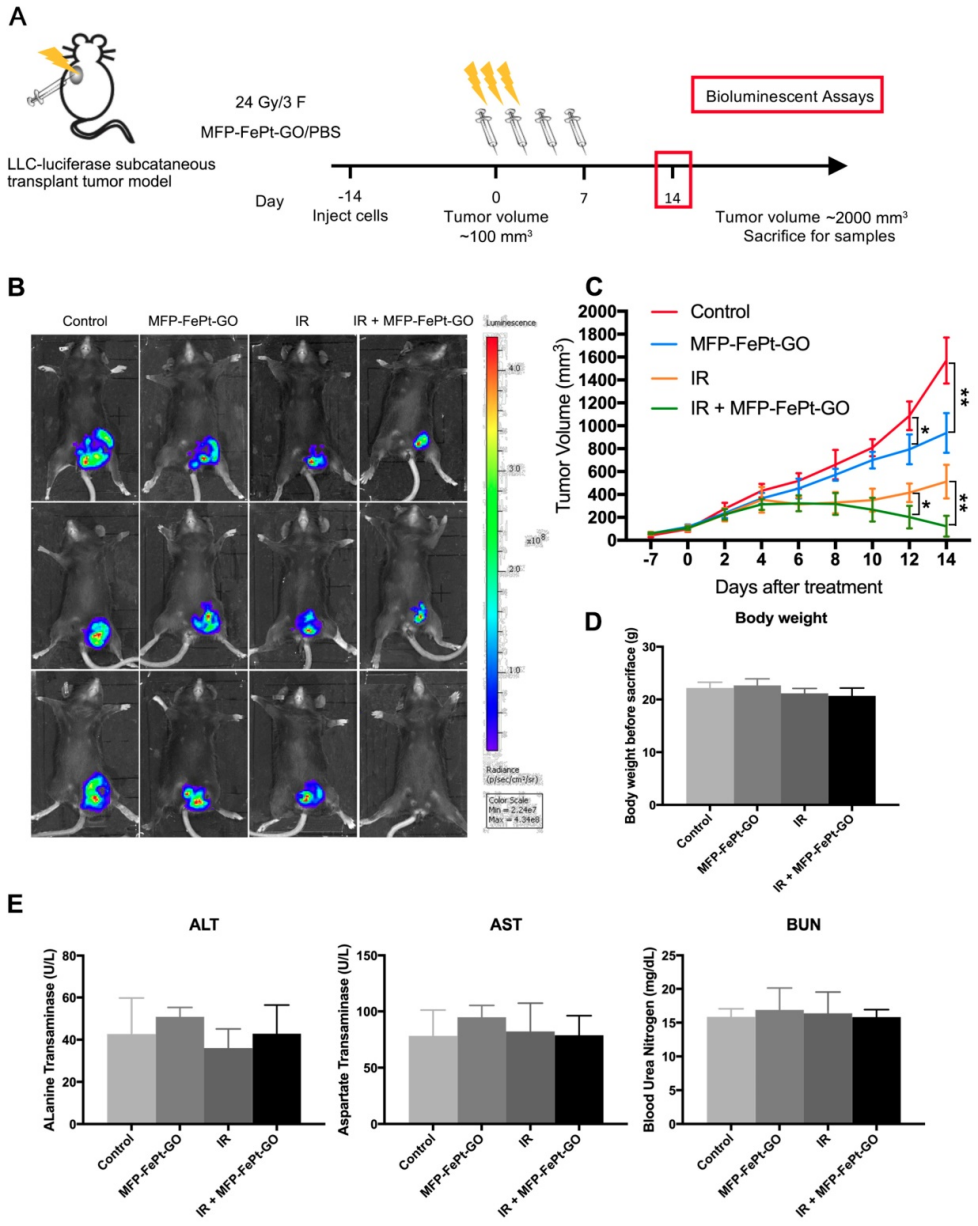
** MFP-FePt-GO inhibits lung tumor growth *in vivo*. (A)** The overall scheme of animal experiments. **(B)** Representative fluorescence images of xenograft tumors *in vivo* show that MFP-FePt-GO has a synergistic effect with radiotherapy to inhibit tumor growth in xenograft lung cancer mouse model. **(C)** Growth curve of tumor volume indicates that the combination therapy significantly inhibits tumor growth *in vivo* compared with single groups.** (D)** Comparison of liver and kidney injury indexes (ALT, AST and BUN) shows no significant difference between groups. Representative images are shown. All values shown were mean ± S.D. of triplicate measurements. *, p < 0.05; **, p < 0.01.

**Figure 7:**
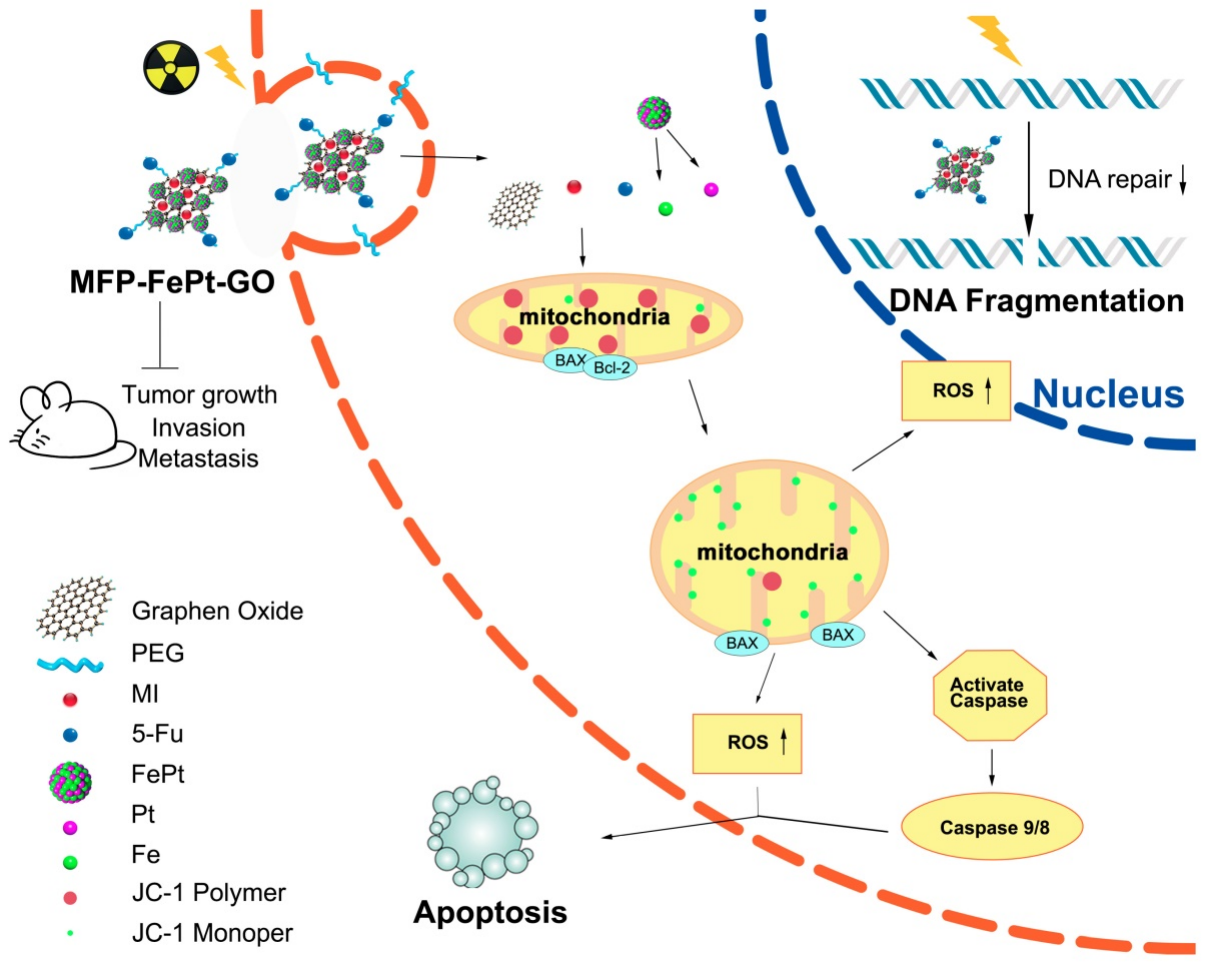
** The whole concept of our experiment**. MFP-FePt-GO nanocomposites are endocytosed by cells, resulting in DNA damage via membrane potential changes of mitochondria and upregulation of ROS level. Accompanied with radiotherapy, it causes more DNA damage and apoptosis. MFP-FePt-GO Nanocomposites combined with radiation therapy *in vivo* can reduce the tumor burden and has the potential of inhibiting tumor cell invasion and metastasis.

We conducted animal experiments according to specific operating procedures(Fig. 6A). MFP-FePt-GO NCs significantly suppressed tumor growth and exerted synergistic effects with radiation. The luciferase-mediated bioluminescence showed that the combination of MFP-FePt-GO NCs and radiotherapy further inhibited tumor growth than individual treatment (Fig. 6B). The tumor volumes between control and MFP-FePt-GO NC groups had a statistically significant difference from the 10^th^ day of treatment, so as the tumor volume between radiation and the combination groups (Fig. 6C). In addition, none of the mice showed clear body weight loss or abnormal symptoms after drug treatments (Fig. 6D). The levels of biochemical indexes (ALT, AST, and BUN) in the serum were used to detect early liver and kidney damage. They were all in the normal range and had no significant difference between groups (Fig. 6E). Collectively, the combination of MFP-FePt-GO NCs and radiation yielded a superior response than that of radiation alone *in vivo*. The whole concept of our experiment was shown in Fig. 7: MFP-FePt-GO Nanocomposites are endocytosed by cells, resulting in DNA damage via membrane potential changes of mitochondria and upregulation of ROS level. Accompanied with radiotherapy, it causes more DNA damage and apoptosis. MFP-FePt-GO Nanocomposites combined with radiation therapy *in vivo* can reduce the tumor burden and has the potential of inhibiting tumor cell invasion and metastasis.

## Discussion

The extraordinary physicochemical properties of graphene-based nanomaterials make them promising tools in nanotechnology and biomedicine 29. In our study, the novel MFP-FePt-GO NCs showed satisfied safety and efficacy as a potential radiosensitive agent for NSCLC patients. Furthermore, we revealed that MFP-FePt-GO NCs promoted radiosensitivity of NSCLC cells via activating intrinsic mitochondrial-mediated apoptosis and impairing DNA damage repair.

This nano drug-delivery system, MFP-FePt-GO NCs, were well designed. The MI was used as the radiosensitizer, while the 5-FU was applied in concurrent chemoradiotherapy. The MI was conjugated to the surface of GO, while the 5-FU was loaded via physisorption with the support of PEG, which implied a different releasing-way in the cellular environment 19. GO was reported to induce a significant mitochondrial membrane depolarization and cellular ROS production in human HaCaT skin keratinocytes 29. This might be mediated by increased cellular ROS production induced by flavoprotein-based oxidative enzymes, such as NADH dehydrogenase and xanthine oxidase. 5-FU converts into effective fluorouracil deoxynucleotides in cells and blocks intracellular deoxyuridylate transformation to deoxy-thymidylate via inhibiting thymidylate synthase, resulting in DNA synthesis interference 30. Our study disclosed the additive effects between MI and 5-FU on chemoradiotherapy. Besides, FePt NPs were reported to be transported into cells by endocytosis and processed by lysosome, producing Fe^2+^ to stress the cell and increase mitochondrial ROS 31. In our study, the MFP-FePt-GO NCs performed as a chemoradiotherapy enhancement agent to reduce the concentration of chemotherapeutic drugs and the dose of radiation. Co-administration of the novel NCs and radiation will reduce the dose and cytotoxicity of radiotherapy in the surrounded normal tissues and organs.

Mitochondria is not only a sensor of endogenous apoptosis pathway, but also an amplifier of apoptosis signals, which can make apoptosis rapid and efficient 32. Loss of mitochondrial electrochemical potential gradient is a typical early event of apoptosis 33. Our data showed that MFP-FePt-GO NCs transformed JC-1 polymer to JC-1 monomer on the mitochondria, suggesting the decrease of the mitochondrial membrane potential in NSCLC cells. The change of ROS level may result from mitochondrial permeability of cells. In fact, we found that MFP-FePt-GO NCs did increase the intracellular ROS levels and induce cell apoptosis, which could be partially inhibited by NAC, which is a typical antioxidant compound 34, indicating MFP-FePt-GO NC treatment activated the intrinsic apoptosis signaling pathway mediated by mitochondria. Therefore, targeting ROS might be an effective therapeutic strategy for cancer management 35.

Radiation causes various DNA damage, such as bases break and cleavage of DNA backbone, including single strand break and double strand break (DSB) 36. In response to DSB, the histone H2AX is phosphorylated at Serine 139 in a region of several mega-base pairs, forming discrete nuclear foci, which can be detectable by immunofluorescence microscopy. Our study demonstrated that radiosensitization of MFP-FePt-GO NCs was manifested by inhibition of DNA damage repair and activation of pro-apoptotic proteins in NSCLC cells. The expression of H2AX was up-regulated, and the expression of DNA damage-inducing protein GADD45 and DSB repair protein RAD51 was downregulated. An undetermined DNA damage signal was reported to induce chromatin remodeling at the GADD45 promoter while cooperating with p53 to activate GADD45 transcription 37. Thus, these results revealed a potential mechanism for the radiosensitive enhancement of MFP-FePt-GO NCs.

The epithelial-mesenchymal transition (EMT) was reported to be involved in the radioresistance and tumor metastasis process^38^. In the current study, our results indicated that tumor cell migration and invasion were suppressed with MFP-FePt-GO NCs treatment by inhibiting EMT. This phenomenon might indicate that MFP-FePt-GO NCs can reverse the radioresistant status of NSCLC cells.

## Conclusion

MFP-FePt-GO NCs inhibited NSCLC tumor growth by inducing cell apoptosis and impairing DNA damage repair possibly via the release of FePt and radiosensitivity chemotherapeutics through a Fenton's reaction in lysosomes. Our animal studies demonstrated the safety and effectiveness of the novel NCs *in vivo*. However, the standards and protocols for nanomaterials administration and regulation issues surrounding nanomaterial translation to clinic need to be further discussed. Further research on the pharmacokinetic and systemic application toxicity of MFP-FePt-GO NCs are still urgently needed. Taken together, our studies revealed the potential of MFP-FePt-GO NCs as a radiation sensitizer to develop a feasible system of novel NCs against NSCLC.

## Supplementary Material

Supplementary figures and tables.

## Abbreviations

NCs: nanocomposites
NSCLC: non-Small cell lung cancer
GO: graphene oxide
NPs: nanoparticles
5-FU: 5-fluorouracil
PEG: polyethylene glycol
MI: 2-(2-methyl-5-nitro-1H-imidazole-1-yl)
TEM: transmission electron microscope
XPS: X-ray photoelectron spectroscopy
EDS: energy-dispersive X-ray spectroscopy
LLC: Lewis lung carcinoma
OD: optical density
PFA: paraformaldehyde
ROS: reactive oxygen species
ALT: alanine transaminase
AST: aspartate aminotransferase
BUN: blood urea nitrogen
NAC: N-acetylcysteine
DSB: double strand break
EMT: epithelial-mesenchymal transition
