# Supplementary material for: MFP-FePt-GO Nanocomposites Promote Radiosensitivity of Non-Small Cell Lung Cancer Via Activating Mitochondrial-Mediated Apoptosis and Impairing DNA Damage Repair
Source: Int J Biol Sci. 2020 May 18;16(12):2145–58. doi: 10.7150/ijbs.46194 (PMC7294941; doi:10.7150/ijbs.46194)
Supplement: Supplementary file 1 — Supplementary figures and tables. [file ijbsv16p2145s1.pdf]

Figure S1

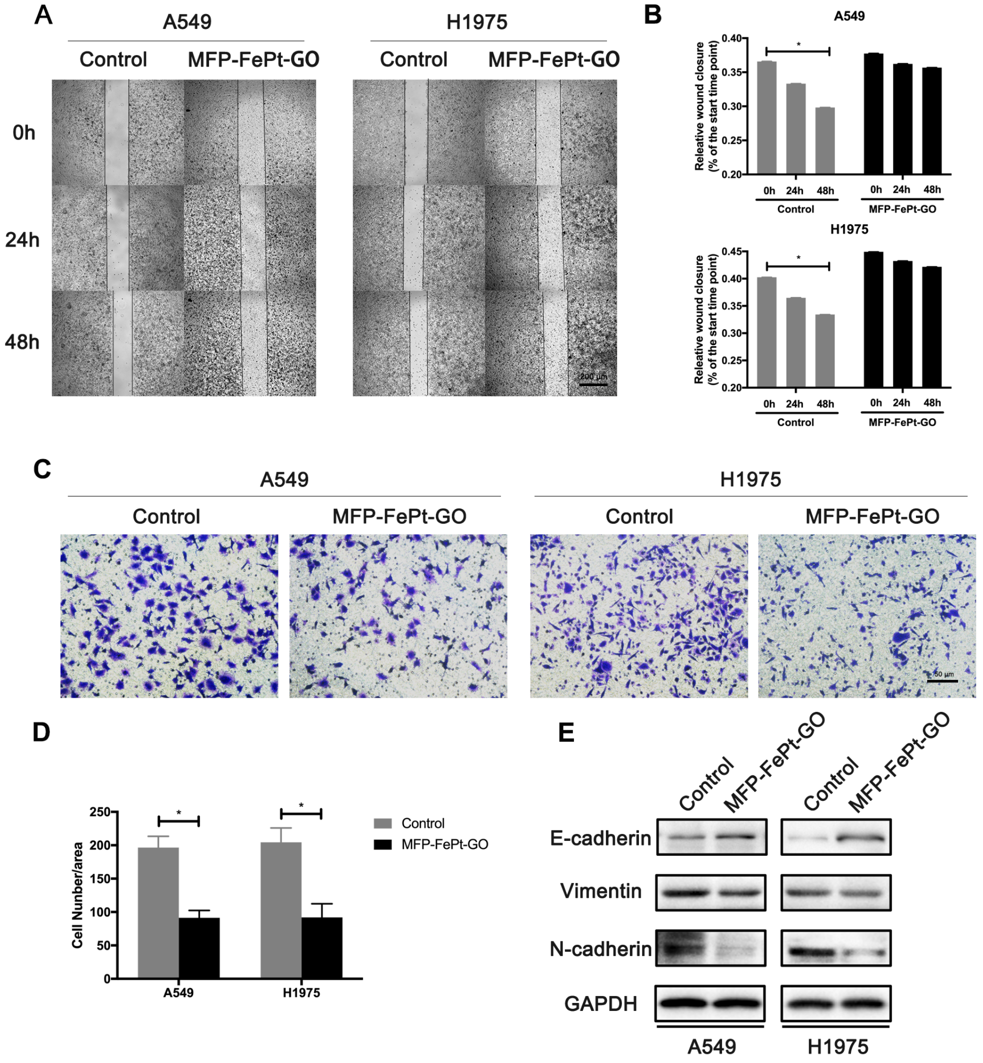

Figure S1. **MFP-FePt-GO suppresses migration and invasion of NSCLS cells. (A)** and **(B)** Wound healing assay shows that the MFP-FePt-GO (20 μg/mL) suppresses NSCLC cell migration. **(C)** and **(D)** Transwell invasion assay shows that MFP-FePt-GO suppresses NSCLC cell invasion. **(E)** Representative immunoblotting of proteins involved in cell migration and EMT. *, $p < 0.05$; **, $p < 0.01$.
